# An 11-gene blood transcriptomic signature reflects a sepsis-associated host-response pattern across public cohorts

**DOI:** 10.3389/fmed.2026.1844619

**Published:** 2026-06-18

**Authors:** Congcong Qin, Weiwei Wang, Qinyuan Du, Tejin Ba, Shuanglin Zhang, Guochen Li

**Affiliations:** 1 Institute of Chinese Medical Literature and Culture, Shandong University of Traditional Chinese Medicine, Jinan, China; 2 Affiliated Hospital of Shandong University of Traditional Chinese Medicine, Jinan, China; 3 Inner Mongolia International Mongolian Hospital, Hohhot, Inner Mongolia, China

**Keywords:** blood transcriptomics, external validation, gene signature, host response, public cohorts, Sepsis

## Abstract

**Background:**

Sepsis is a clinically defined syndrome caused by a dysregulated host response to infection, yet the biological architecture underlying this response remains highly heterogeneous. Blood transcriptomic studies have shown that patients with sepsis can be subdivided into reproducible molecular states; however, translation has been hindered by inconsistent gene sets, cohort-specific modeling strategies, and insufficient external validation across public datasets. We aimed to derive a compact blood transcriptomic signature to characterize a sepsis-associated host-response pattern and to evaluate its portability across independent public cohorts and clinically distinct validation settings.

**Methods:**

Using GSE65682 as the discovery cohort, we identified differentially expressed genes in the Abdominal_Sepsis vs. GI_Control comparison, prioritized hub genes through STRING-PPI analysis, and entered the top 20 degree-ranked candidates into cross-validated LASSO-logistic modeling. The final 11-gene signature was defined according to the nonzero coefficient rule. Because the discovery coefficients were estimated from standardized variables, external fixed-score evaluation was performed after the retained genes were transformed using cohort-specific StandardScaler procedures within each external analysis cohort. External analyses were performed using GSE236713 across three Day 1 settings: OOHCA-SIRS vs. sepsis, abdominal vs. pulmonary sepsis, and survival among patients with sepsis. GSE54514 was used as a supplementary, non-confirmatory cohort.

**Results:**

In GSE236713, the standardized fixed-coefficient score showed moderate discrimination between Day 1 OOHCA-SIRS and sepsis (AUC = 0.7676; Mann–Whitney *P* = 4.74 × 10^−7^), whereas the abdominal vs. pulmonary sepsis and Day 1 survival analyses showed near-null discrimination (AUC = 0.5126 and 0.5291; *P* = 0.8119 and 0.6334, respectively). GSE54514 was retained as a supplementary, non-confirmatory cohort.

**Conclusions:**

We identified an 11-gene blood transcriptomic score that may reflect a sepsis-associated host-response pattern. The score showed moderate discrimination between Day 1 sepsis and OOHCA-SIRS, whereas the available Day 1 analyses did not demonstrate clear discrimination by infection source or short-term survival under the current analysis settings.

## Introduction

Sepsis remains one of the most challenging syndromes in critical care because its clinical definition successfully captures high-risk patients while simultaneously obscuring major biological diversity (1, 2). At the bedside, patients who satisfy the same diagnostic framework may differ markedly in inflammatory amplitude, myeloid cell mobilization, lymphocyte dysfunction, endothelial injury, coagulopathic tone, tissue hypoperfusion, and recovery trajectory (3, 4). This disconnect between clinical recognition and biological granularity has led to limited progress in precision therapeutics. Therapeutic failure in unselected sepsis populations is now understood not simply as a problem of inadequate drug selection, but as a consequence of treating biologically heterogeneous patients as if they represented a single inflammatory entity (5). In this context, molecular stratification has emerged as more than a descriptive exercise. It has become a central requirement for identifying reproducible host states, understanding the mechanistic basis of clinical variability, and designing interventions targeted at biologically appropriate populations (6).

### Blood transcriptomics as the most deployable systems-level window into sepsis biology

Among the available omics approaches, blood transcriptomics is uniquely positioned at the interface between discovery biology and clinical translatability. Whole-blood and leukocyte transcriptomes can be obtained from routine samples, preserve broad immune-state information, and have repeatedly shown that sepsis contains a coherent molecular structure rather than irreducible noise (7, 8). Prior work has demonstrated that patients can be assigned to transcriptomic response classes characterized by varying combinations of granulocytic activation, adaptive immune suppression, antigen presentation impairment, endothelial stress, coagulation pathway engagement, and metabolic remodeling (8–11). This literature has shifted the field away from the oversimplified notion of sepsis as a uniform cytokine storm and toward a model in which multiple host-response programs coexist and vary in dominance across patients and over time (7–12). However, the richness of these transcriptomic models has also created a translational bottleneck, as the most biologically informative systems are often the least portable across cohorts and platforms (8, 10, 12).

### The unmet need for a compact and portable host-response signature

A major unresolved problem in sepsis transcriptomics is how to translate complex endotype frameworks into forms that are both biologically interpretable and feasible for external use (6, 8, 12). Large endotype systems have substantially advanced mechanistic understanding; however, many remain difficult to implement outside their original analytic environment because they depend on high-dimensional classifiers, cohort-specific preprocessing, or extensive gene panels (6, 8, 12). As a result, biological richness does not automatically translate into portability or practical deployability across independent cohorts.

In practice, clinical and translational studies often require a lower-dimensional index that characterizes a sepsis-associated host-response pattern without attempting to summarize the full spectrum of disease heterogeneity (13–16). Such a signature is not expected to explain every axis of sepsis biology. Its value lies instead in providing a reproducible, calculable, and externally testable representation of a common immunobiological state that can be transferred across datasets (14–17). A compact signature of this kind may therefore serve as a bridge between large-scale discovery frameworks and future assay-oriented translation. Recent transcriptomic studies in other complex diseases have similarly shown that reduced and interpretable gene panels can improve translational deployability while retaining biological or prognostic relevance (18–20).

### Why multi-scenario external validation is more informative than single-scenario comparisons

External validation in sepsis transcriptomics should not be treated as a purely binary exercise of success or failure. The biological meaning of a signature depends strongly on the comparison context in which it is tested. A marker that performs well against healthy controls may mainly reflect nonspecific illness-related disturbance, whereas a marker that distinguishes sepsis from sterile systemic inflammation is more likely to reflect an infection-associated host-response pattern (13, 17).

Different validation settings can therefore imply different kinds of biological meaning. A marker that separates distinct infectious or inflammatory contexts may reflect setting-specific biology, whereas a marker that predicts severity or early mortality may represent a prognostic axis rather than a disease-state signature (14–16). These distinctions matter because the scientific and translational value of a biomarker depends not only on whether it works but also on what it actually measures. Therefore, we treated external validation not only as a confirmation of discriminative ability but also as a framework for defining the boundary of interpretation (15–17).

### Study objective and conceptual hypothesis

The present study was designed to derive a compact 11-gene blood transcriptomic score to characterize a sepsis-associated host-response pattern and to evaluate its interpretive positioning across multiple independent public cohorts (6, 12, 13, 17). Using GSE65682 as the discovery dataset, we derived the weighted 11-gene score through a disease-comparison–anchored DEG, STRING-PPI, and LASSO-logistic workflow and subsequently interpreted the resulting score in relation to the internal host-response subtype structure and subtype-associated feature distributions. We then evaluated this score in GSE236713 in three deliberately distinct Day 1 settings: OOHCA-SIRS vs. sepsis, abdominal vs. pulmonary sepsis, and survival among patients with sepsis. GSE54514 served as an additional supplementary, non-confirmatory cohort. Our central hypothesis was that the resulting signature would primarily reflect a sepsis-associated host-response pattern rather than function as a source-specific or short-term outcome-specific classifier (6, 13, 17).

## Materials and methods

### Overall study design and analytical strategy

This study was conducted as a public-cohort-based discovery and external evaluation study focused on deriving a compact blood transcriptomic signature with clear biological anchoring. The analytical framework contained four linked layers. First, the discovery layer used preprocessed Day 1 data from GSE65682 to identify and interpret the host-response subtype structure. Second, a reduced gene signature was constructed from the GSE65682 Abdominal_Sepsis vs. GI_Control differential-expression contrast, followed by STRING-based PPI hub-gene prioritization and cross-validated LASSO-logistic coefficient estimation. The discovery-layer subtype framework was then used as an internal biological interpretation layer to evaluate subtype-level expression patterns and coefficient–expression relationships. Third, the primary external analysis layer used GSE236713 to test the score in multiple Day 1 clinical settings selected to define the biological boundary of the signature. Fourth, GSE54514 was used as a supplementary, non-confirmatory external dataset to assess signal consistency and interpretive boundaries. This stepwise design was chosen because it prioritizes interpretability and portability over single-comparison optimization. Comparable studies in sepsis transcriptomics have similarly used multicohort aggregation, subtype anchoring, and externally applied signatures to clarify both biological meaning and translational relevance (6, 8, 9, 12, 13, 17).

### Discovery cohort selection and internal subtype framework

The discovery cohort was based on GSE65682. Subtype labels in GSE65682 were generated within the present discovery workflow rather than imported from an external classifier. Briefly, 93 samples retained for the GSE65682 discovery-layer subtype analysis were represented by a fixed 13-feature host-response matrix. The feature matrix was z-score–normalized using StandardScaler prior to clustering. K-means clustering was then applied to this fixed feature matrix, with candidate *k*-values from 2 to 6 evaluated using silhouette score, inertia, cluster balance, Calinski–Harabasz index, and Davies–Bouldin index. For *k* = 2, the silhouette score was 0.3678 and the inertia was 675.5723, yielding cluster sizes of 50 and 43. After label harmonization, the resulting assignments defined Subtype_1 (*n* = 43) and Subtype_2 (*n* = 50). The complete feature list and k-selection metrics are provided in Supplementary Tables 1, 2. These fixed discovery-layer subtype labels were used to support internal biological interpretation and subtype-level expression-direction analysis, but they were not used as the binary outcome for LASSO-logistic coefficient estimation. External cohorts were not reclustered.

### Differential-expression evidence and STRING-PPI hub prioritization

For signature construction, differential-expression analysis was performed in GSE65682 using the Abdominal_Sepsis vs. GI_Control comparison. Significant differentially expressed genes were defined by Benjamini–Hochberg adjusted *P*-value < 0.05 and absolute log_2_ fold change ≥ 1.0. This criterion yielded 304 significant genes, which were used as the DEG-supported input for STRING-PPI analysis.

STRING-based protein–protein interaction analysis was performed using the STRING network API for Homo sapiens (species 9,606) with required_score = 400. Genes mapped to the STRING-derived interaction network were ranked according to node degree, with weighted degree retained as a secondary network quantity. The top 20 degree-ranked hub genes were carried forward as PPI-prioritized candidate genes for LASSO-logistic modeling. The PPI-prioritized candidate genes were not directly treated as the final signature; instead, they were passed to penalized logistic modeling for coefficient estimation and final gene retention.

In parallel, subtype-associated expression statistics were used for internal biological interpretation of the discovery-layer host-response structure. For this subtype-level analysis, expression direction was defined by the mean expression difference between Subtype_2 and Subtype_1, with positive values indicating higher mean expression in Subtype_2 and negative values indicating higher mean expression in Subtype_1.

### LASSO-logistic coefficient estimation and final 11-gene retention

The top 20 degree-ranked STRING-PPI hub genes were entered into a LASSO-logistic regression model using a predefined binary modeling label, in which Abdominal_Sepsis was coded as 1 and GI_Control was coded as 0. Before model fitting, candidate gene expression values were standardized using StandardScaler. The model was implemented using LogisticRegressionCV with an L1 penalty, the saga solver, 5-fold stratified cross-validation through StratifiedKFold, roc_auc scoring, random_state = 42, and max_iter = 5,000. Genes with nonzero coefficients in the final cross-validated LASSO-logistic model were retained as the final signature genes. This nonzero coefficient rule was used as the definitive criterion for final gene selection after DEG-supported and STRING-PPI-based candidate prioritization.

This procedure yielded 11 retained genes: LTF, GZMB, IL1B, MPO, TBX21, HLA-DRB1, GZMK, HLA-DRA, PRF1, CD8A, and CCR7. The corresponding coefficients were derived from the discovery-layer LASSO-logistic model and were fixed before external evaluation.

### Signature score computation and coefficient preservation

Before LASSO-logistic model fitting, candidate gene expression values in the GSE65682 discovery modeling matrix were centered and scaled using StandardScaler. The retained coefficients therefore reflect standardized gene-expression variables. For external fixed-score evaluation, probe annotation was cleaned and expression values were reconstructed at the gene level within each external cohort. The expression values of the 11 retained genes were then centered and scaled within each external analysis cohort before multiplication by the fixed discovery-derived coefficients. The resulting standardized fixed-coefficient score was used for ROC analysis, Mann–Whitney U testing, and visualization. No external cohort was used for subtype reclustering, feature reselection, coefficient re-estimation, or model refitting.

For GSE236713, after gene-level reconstruction, the 11 retained signature genes were centered and scaled within the GSE236713 Day 1 analysis matrix using StandardScaler. The standardized expression values were multiplied by the fixed discovery-derived coefficients to generate the external standardized signature score. The same standardized score table was then used for the OOHCA-SIRS vs. sepsis comparison, the abdominal vs. pulmonary sepsis comparison, and the survival analysis among patients with sepsis.

For GSE54514, after gene-level reconstruction, the 11 retained genes were centered and scaled within the GSE54514 analysis matrix before fixed-coefficient score calculation, using the same external fixed-score convention as in GSE236713.

In the following formula, Z(gene) denotes the cohort-specific standardized expression value of the corresponding gene in the analyzed cohort: Score = 0.970126 × Z(LTF) – 0.728580 × Z(GZMB) – 0.592121 × Z(IL1B) + 0.542930 × Z(MPO) – 0.494194 × Z(TBX21) – 0.474606 × Z(HLA-DRB1) + 0.385883 × Z(GZMK) – 0.359247 × Z(HLA-DRA) – 0.101235 × Z(PRF1) + 0.051392 × Z(CD8A) – 0.014266 × Z(CCR7).

The full fixed-coefficient values are reported in Table 1. In external datasets, the score was calculated after cohort-specific probe annotation cleaning, gene-level reconstruction, and cohort-specific standardization of the 11 retained genes. Thus, GSE236713 and GSE54514 were used to evaluate the transferability and interpretive boundary of the fixed discovery-derived score rather than to retrain the signature.

**Table 1:** Fixed 11-gene signature coefficients and subtype-level expression directions in the discovery cohort.

Gene	Coefficient	Absolute coefficient	Coefficient direction	Subtype-level expression direction	Coefficient–expression relation	Interpretation note
LTF	0.9701255021549925	0.9701255021549925	Positive	Up	Same apparent direction	Coefficient sign represents mathematical contribution to the fixed signature score.
GZMB	−0.7285803292808798	0.7285803292808798	Negative	Down	Same apparent direction	Coefficient sign represents mathematical contribution to the fixed signature score.
IL1B	−0.5921212968857412	0.5921212968857412	Negative	Down	Same apparent direction	Coefficient sign represents mathematical contribution to the fixed signature score.
MPO	0.5429302767734766	0.5429302767734766	Positive	Up	Same apparent direction	Coefficient sign represents mathematical contribution to the fixed signature score.
TBX21	−0.49419352560731145	0.49419352560731145	Negative	Down	Same apparent direction	Coefficient sign represents mathematical contribution to the fixed signature score.
HLA-DRB1	−0.474606010714216	0.474606010714216	negative	Down	Same apparent direction	Coefficient sign represents mathematical contribution to the fixed signature score.
GZMK	0.38588325522540484	0.38588325522540484	Positive	Down	opposite apparent direction	Coefficient direction should not be equated with inter-group expression direction.
HLA-DRA	−0.35924678677700733	0.35924678677700733	Negative	Down	Same apparent direction	Coefficient sign represents mathematical contribution to the fixed signature score.
PRF1	−0.10123530433072744	0.10123530433072744	Negative	Down	Same apparent direction	Coefficient sign represents mathematical contribution to the fixed signature score.
CD8A	0.05139156506430512	0.05139156506430512	Positive	Down	opposite apparent direction	Coefficient direction should not be equated with inter-group expression direction.
CCR7	−0.014266291692989385	0.014266291692989385	Negative	Down	Same apparent direction	Coefficient sign represents mathematical contribution to the fixed signature score.

Coefficient direction and subtype-level expression direction represent distinct quantities. Coefficient direction indicates the mathematical contribution of each gene to the fixed multivariable LASSO-derived score after joint modeling, whereas subtype-level expression direction indicates the observed mean expression difference between Subtype_2 and Subtype_1 in the discovery-layer comparison. Therefore, coefficient sign should not be interpreted as direct evidence of upregulation or downregulation. GZMK and CD8A had positive coefficients but lower mean expression in Subtype_2 than in Subtype_1, indicating an opposite coefficient–expression relation that requires separate interpretation.

The coefficient sign and the subtype-level expression direction represent distinct quantities and were interpreted accordingly. The coefficient signs were derived from the Abdominal_Sepsis vs. GI_Control LASSO-logistic model, whereas subtype-level expression directions were derived from the Subtype_2 vs. Subtype_1 discovery-layer expression comparison; these quantities were therefore treated as non-interchangeable.

A positive or negative coefficient indicates the mathematical contribution of a gene to the fixed multivariable score after joint penalized modeling, whereas subtype-level expression direction indicates the observed mean expression difference between Subtype_2 and Subtype_1 in the discovery-layer comparison. Therefore, coefficient direction was not used as a direct synonym for upregulation or downregulation. This distinction was particularly important for GZMK and CD8A, both of which carried positive coefficients in the fixed score but showed lower mean expression in Subtype_2 than in Subtype_1. The fixed-coefficient score was therefore interpreted as a multivariable mathematical summary of the discovery-derived host-response pattern, not as a single-gene expression-direction scale.

### Internal biological interpretation of the signature score

To evaluate whether the score retained biological meaning within the discovery cohort, we interpreted it in the context of the confirmed 13-feature host-response matrix used for subtype discovery. This matrix summarized subtype-discriminative host-response feature scores, with the complete feature definitions provided in Supplementary Table 1. The goal of this step was not only to confirm that the score differed across the two subtypes but also to test whether it was coherently embedded in a broader host-response framework. A compact signature intended to characterize a sepsis-associated host-response pattern should align with multiple immunobiological transitions simultaneously rather than simply reflect the dominance of one pathway. Therefore, we used the internal feature framework as a biological consistency check. Similar approaches have been applied in recent transcriptomic studies to distinguish reduced signatures that preserve mechanistic coherence from those that achieve discrimination only by overfitting local statistical contrasts (8, 9, 12, 15).

### Selection of external analysis cohorts

External cohorts were chosen according to four criteria: phenotype annotation clarity, availability of Day 1 or baseline samples, feasibility of gene-level harmonization, and capacity to test distinct biological questions. GSE236713 was selected as the primary external analysis cohort because it includes OOHCA-SIRS, abdominal sepsis, pulmonary sepsis, and survival-related annotations, allowing the same signature to be tested under multiple clinically meaningful comparison structures within one dataset. GSE54514 was retained as a supplementary, non-confirmatory external cohort because it includes healthy controls and prognosis-related groupings, albeit with a smaller and more uneven sample structure. GSE154918 was downloaded, parsed, and explored as a candidate external resource. However, it was not incorporated into the primary validation framework because a reproducible linkage between the expression matrix and phenotype annotation could not be established with sufficient stability for the main analysis. This reflects a common challenge in public data–based biomarker studies, where analytic availability and analytic usability are not equivalent. Cohort selection strategies emphasizing annotation quality and biological interpretability have been repeatedly highlighted as essential in cross-cohort sepsis transcriptomics (8, 9, 12, 21).

### Construction of comparison groups in GSE236713

Within GSE236713, sample metadata were parsed to define disease group, timepoint, and outcome variables. Three Day 1 comparison files were then constructed. The first compared OOHCA-SIRS with sepsis and was intended to test whether the 11-gene signature could distinguish infection-associated dysregulated host responses from severe but noninfectious inflammatory illnesses. The second compared abdominal sepsis with pulmonary sepsis and was designed to determine whether the signature primarily encoded the infection source. The third compared survivors with nonsurvivors among patients with sepsis and was used to assess whether the signature behaved as a direct short-term prognostic signal. By evaluating all three scenarios within the same external cohort, we sought to define the interpretive boundary of the score. This boundary-oriented approach is more informative than reporting only one favorable comparison because it clarifies not only where the signature performs well but also where its biological meaning appears limited. Such boundary-oriented validation is increasingly recognized as necessary for responsible biomarker interpretation in heterogeneous syndromes such as sepsis (15, 17, 21, 22).

### Gene-level expression harmonization for GSE236713

Because GSE236713 is a microarray-based dataset, the GEO series matrix and the corresponding platform annotation were parsed to construct a cohort-specific gene-level expression matrix. The expression values were inspected on the imported analysis scale, and log_2_ transformation was not reapplied because the imported matrix was already on a comparable log-transformed scale. Probe identifiers were mapped to official gene symbols according to the corresponding GPL annotation file. Probes without valid gene symbols and probes mapping ambiguously to multiple symbols were excluded. When multiple uniquely mapped probes corresponded to the same gene symbol, their values were averaged on the analysis scale to generate one expression value per gene within the cohort. After harmonization, 32,080 gene-level entries were retained, and all 11 signature genes were successfully mapped. After gene-level reconstruction, the 11 retained signature genes were centered and scaled within the GSE236713 Day 1 analysis matrix using StandardScaler. The standardized expression values were multiplied by the fixed discovery-derived coefficients to generate the external standardized signature score. The same standardized score table was then used for the OOHCA-SIRS vs. sepsis comparison, the abdominal vs. pulmonary sepsis comparison, and the survival analysis among patients with sepsis. Heatmaps used row-wise z-score expression to display gene-level patterns and were not treated as standardized signature scores.

### Supplementary non-confirmatory analysis strategy in GSE54514

GSE54514 was analyzed as a supplementary, non-confirmatory cohort to assess directional support and interpretive boundaries. The GEO series matrix and the corresponding annotation resources were parsed to derive Day 1 control vs. sepsis and Day 1 survivor vs. nonsurvivor groupings. Gene-level reconstruction followed the same overall principle as in the primary external cohort: probe annotation was cleaned at the cohort level, invalid or ambiguous mappings were excluded, and when multiple uniquely mapped probes corresponded to the same gene symbol, their values were averaged on the analysis scale to generate one gene-level expression value per symbol. After gene-level reconstruction in GSE54514, the 11 retained genes were centered and scaled within the GSE54514 analysis matrix before fixed-coefficient score calculation, using the same external fixed-score convention as in GSE236713. However, GSE54514 was not treated as a confirmatory validation resource, given its smaller sample size, less uniform temporal structure, and less favorable group balance. This supplementary analysis was therefore interpreted as non-confirmatory evidence for defining interpretive boundaries rather than as a stand-alone benchmark of external performance.

### Statistical analysis and presentation strategy

The primary variable for between-group comparisons in the external analyses was the sample-level standardized fixed-coefficient transcriptomic signature score. All external analyses were conducted under a uniform framework centered on this score. Because the external comparisons involved independent public cohorts or cohort-defined subgroups with unequal sample sizes, normality of the score distributions was not assumed *a priori* across all settings. Score distributions were visually inspected during analysis. Given the unequal and sometimes limited subgroup sizes across external cohort settings, formal normality testing was not considered a sufficiently stable or informative universal decision criterion. We therefore adopted a nonparametric framework, as consistent normality could not be assumed across cohorts and comparison settings.

Between-group differences in the signature score were assessed using the Mann–Whitney *U*-test as a nonparametric comparison of score distributions. Receiver operating characteristic analysis was used to summarize the ability of the continuous signature score to discriminate between predefined binary comparison groups, and the area under the curve was interpreted as a discrimination measure rather than as a fully adjusted predictive modeling result. Because ROC analysis evaluates the ranking-based discrimination of a continuous marker against binary class labels, it does not require a Gaussian distribution assumption for the score itself.

In the discovery layer, STRING-PPI hub genes were ranked by node degree, whereas subtype-level expression summaries were interpreted using mean expression direction and false discovery rate-based significance measures. Heatmaps, boxplots, and other descriptive visualizations were used to display the within-cohort score or gene-pattern structure and were not treated as stand-alone inferential analyses. Heatmaps were constructed after row-wise z-score standardization to emphasize relative cross-group patterns rather than absolute expression levels. Because the objective of the study was to define the biological role and validation boundary of a compact host-response signature rather than to maximize prognostic performance through complex model stacking, we did not perform secondary machine-learning optimization or large covariate-adjusted prognostic modeling. This decision preserved transparency and allowed the same score to be tested comparably across multiple external scenarios. A sensitivity comparison between raw-expression weighted scoring and standardized fixed-coefficient scoring is provided in Supplementary Table 5.

### Software environment

All data processing, statistical analyses, and figure generation in the present study were conducted in a Python-based workflow. The final reproducible analysis environment used Python 3.10.11, with core packages including pandas 2.3.3, numpy 1.26.4, scipy 1.15.3, scikit-learn 1.7.2, and matplotlib 3.10.8. Additional package version information for the final analysis environment is summarized in Supplementary Table 4.

## Results

### Discovery-layer projection revealed a structured host-response continuum

**Figure 1:**
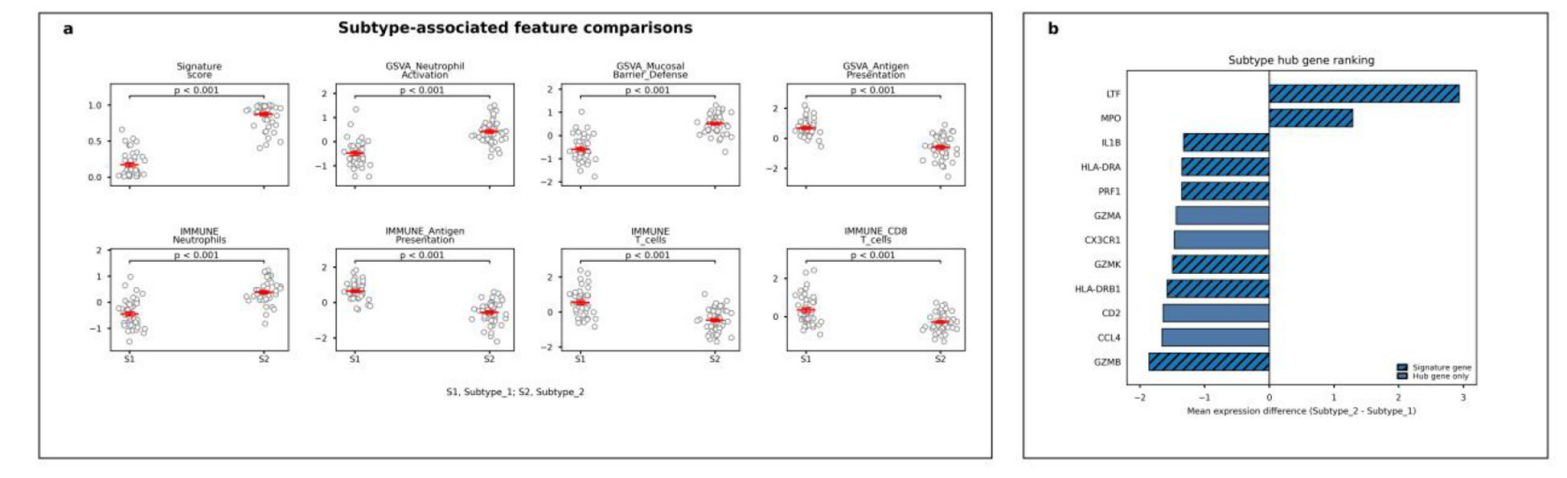
Internal support for the 11-gene blood transcriptomic signature in the discovery cohort. **(a)** Distribution of subtype-associated features across Subtype_1 and Subtype_2 in GSE65682, including the signature score and representative immune or functional features derived from the subtype framework. **(b)** Subtype-specific expression difference ranking among core PPI-prioritized candidate genes. Genes overlapping with the final 11-gene signature are highlighted. Positive values indicate higher mean expression in Subtype_2, whereas negative values indicate higher mean expression in Subtype_1. This panel displays subtype-level expression-direction patterns rather than LASSO coefficient directions. GSVA, gene set variation analysis; FDR, false discovery rate.

In the discovery cohort, the 11-gene signature score aligned closely with the discovery-layer host-response subtype structure. When projected onto the Day 1 subtype assignments, a clear separation between Subtype_1 and Subtype_2 was evident, consistent with preservation of the dominant biological contrast embedded in the discovery data. This difference was not restricted to a small subset of extreme samples and was not driven by one outlier gene. Instead, it reflected a broader shift in the internal host-response landscape. Samples with higher scores tended to cluster with stronger neutrophil-associated and inflammatory–myeloid features, whereas those with lower scores aligned more closely with patterns reflecting relative preservation of antigen presentation and lymphoid activity. These observations support the interpretation that the score functions as a reduced representation of a host-response continuum rather than merely a substitute for a discrete subtype label. Together, these discovery-layer observations provided initial internal evidence that the 11-gene signature retained biologically structured information and could serve as a compressed state index rather than a dataset-specific classifier. Figure 1 summarizes the internal support for the 11-gene signature in the discovery cohort by displaying subtype-associated feature distributions together with the core hub-gene ranking pattern. This discovery-layer separation was interpreted as internal biological consistency rather than as independent validation of the score, because the 13-feature matrix included an internal signature-score dimension. The fixed 11-gene coefficients were generated through the Abdominal_Sepsis vs. GI_Control DEG–PPI–LASSO workflow, and the standardized fixed-coefficient score was then applied to external cohorts without refitting.

### PPI-prioritized candidate genes and expression statistics clarified the multigene structure underlying the score

Examination of the PPI-prioritized candidate genes and their subtype-specific expression statistics provided a more detailed view of the biological structure underlying the score. LTF and MPO showed higher mean expression in Subtype_2 than in Subtype_1, indicating a granulocyte- or myeloid-associated component in the discovery-layer contrast. In contrast, several genes related to cytotoxic lymphocyte activity, T-cell-associated programs, and antigen presentation, including GZMB, HLA-DRB1, GZMK, PRF1, HLA-DRA, IL1B, TBX21, CCR7, and CD8A, showed lower mean expression in Subtype_2 than in Subtype_1 in the discovery-layer expression comparison.

However, these expression-direction patterns should be distinguished from LASSO coefficient signs. Coefficient direction represents each gene's mathematical contribution to the fixed multivariable score after joint penalized modeling, whereas subtype-level expression direction represents the observed mean expression difference between Subtype_2 and Subtype_1. This distinction was particularly relevant for GZMK and CD8A, which showed lower mean expression in Subtype_2 but carried positive coefficients in the fixed score. Therefore, the final signature was interpreted as a coordinated multigene state index rather than as a simple directional expression scale.

The final reduced signature consisted of 11 genes whose coefficients were preserved from the discovery-layer LASSO-logistic model. These coefficients were used as fixed multivariable scoring weights and were not interpreted as direct markers of subtype-specific upregulation or downregulation. To clarify the coefficient structure and subtype-associated expression patterns underlying the final score, we summarized the fixed coefficients and subtype-specific expression statistics in Tables 1, 2. Accordingly, Table 2 was used to display subtype-level expression statistics, whereas Table 1 was used to display the fixed LASSO-derived coefficients and their coefficient–expression relationships.

**Table 2:** Subtype-specific expression statistics for core PPI-prioritized candidate genes in the discovery cohort.

Gene_symbol	Subtype_1_n	Subtype_2_n	Subtype_1_mean	Subtype_2_mean	Subtype_1_median	Subtype_2_median	Mean_difference	Abs_mean_difference	*p*_value	In_signature	Direction	FDR
LTF	43.000	50.000	4.885	7.824	4.671	8.091	2.938	2.938	0.000	1.000	Higher_in_Subtype_2	0.000
GZMB	43.000	50.000	6.544	4.688	6.522	4.367	−1.855	1.855	0.000	1.000	Higher_in_Subtype_1	0.000
HLA-DRB1	43.000	50.000	8.768	7.190	8.749	7.176	−1.577	1.577	0.000	1.000	Higher_in_Subtype_1	0.000
GZMK	43.000	50.000	4.935	3.442	4.754	3.254	−1.493	1.493	0.000	1.000	Higher_in_Subtype_1	0.000
PRF1	43.000	50.000	6.063	4.712	6.140	4.526	−1.351	1.351	0.000	1.000	Higher_in_Subtype_1	0.000
HLA-DRA	43.000	50.000	10.396	9.048	10.424	9.025	−1.348	1.348	0.000	1.000	Higher_in_Subtype_1	0.000
IL1B	43.000	50.000	7.558	6.235	7.654	6.262	−1.323	1.323	0.000	1.000	Higher_in_Subtype_1	0.000
MPO	43.000	50.000	3.604	4.895	3.419	4.192	1.291	1.291	0.000	1.000	Higher_in_Subtype_2	0.000
TBX21	43.000	50.000	5.380	4.221	5.352	4.186	−1.159	1.159	0.000	1.000	Higher_in_Subtype_1	0.000
CCR7	43.000	50.000	7.042	5.936	7.066	6.181	−1.105	1.105	0.000	1.000	Higher_in_Subtype_1	0.000
CD8A	43.000	50.000	4.481	3.541	4.607	3.469	−0.940	0.940	0.000	1.000	Higher_in_Subtype_1	0.000
CCL4	43.000	50.000	5.880	4.223	5.885	3.984	−1.657	1.657	0.000	0.000	Higher_in_Subtype_1	0.000
CD2	43.000	50.000	6.324	4.685	6.180	4.610	−1.639	1.639	0.000	0.000	Higher_in_Subtype_1	0.000
CX3CR1	43.000	50.000	8.553	7.085	8.600	7.263	−1.468	1.468	0.000	0.000	Higher_in_Subtype_1	0.000
GZMA	43.000	50.000	6.923	5.485	6.518	5.294	−1.438	1.438	0.000	0.000	Higher_in_Subtype_1	0.000
IL7R	43.000	50.000	6.720	5.462	6.674	5.620	−1.259	1.259	0.000	0.000	Higher_in_Subtype_1	0.000
CD74	43.000	50.000	7.070	5.822	6.937	5.874	−1.248	1.248	0.000	0.000	Higher_in_Subtype_1	0.000
CD3D	43.000	50.000	5.500	4.296	5.516	4.186	−1.204	1.204	0.000	0.000	Higher_in_Subtype_1	0.000
FCGR3A	43.000	50.000	9.375	8.248	9.389	8.420	−1.127	1.127	0.000	0.000	Higher_in_Subtype_1	0.000
LCK	43.000	50.000	4.486	3.369	4.293	3.228	−1.117	1.117	0.000	0.000	Higher_in_Subtype_1	0.000

This table summarizes subtype-specific expression statistics for core PPI-prioritized candidate genes in GSE65682. Positive mean differences indicate higher mean expression in Subtype_2, whereas negative mean differences indicate higher mean expression in Subtype_1. The table displays subtype-level expression patterns and final signature membership; STRING-PPI node-degree prioritization and stepwise candidate-selection rules are summarized in Supplementary Table 3.

### The discovery heatmap confirmed that the signature was network-supported rather than marker-driven

**Figure 2:**
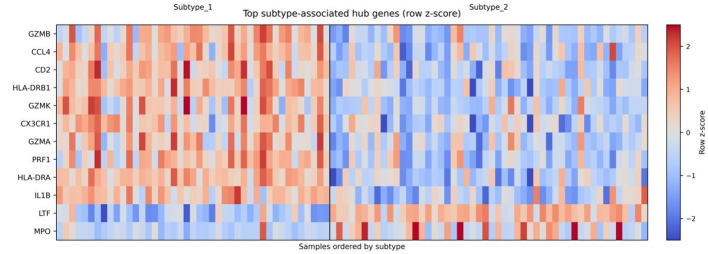
Heatmap of the 11-gene signature across host-response subtypes in the discovery cohort. Heatmap showing row-scaled expression of the 11-gene signature in GSE65682 after ordering samples by subtype. Warmer colors indicate relatively higher expression, and cooler colors indicate relatively lower expression within each gene. The pattern illustrates the coordinated separation of subtype-associated myeloid–inflammatory and lymphoid or antigen presentation–related programs. The heatmap displays relative subtype-level expression patterns and should not be interpreted as a direct visualization of LASSO coefficient signs. z-score, within-gene standardized expression value.

The discovery-layer heatmap further demonstrated that the 11-gene signature represented an organized multigene program rather than dependence on a single dominant marker. After samples were reordered according to subtype, a coherent expression structure became apparent. LTF and MPO were relatively enriched toward the Subtype_2 side of the sample spectrum, whereas several genes related to lymphoid or antigen-presentation programs were relatively enriched toward the Subtype_1 side. This visual pattern was interpreted as a subtype-level expression structure and was not used to infer the direction of individual LASSO coefficients. Although individual sample variation remained, the general pattern was highly interpretable and directionally stable. This observation is relevant because transcriptomic signatures built on one or two dominant features may show reduced reproducibility in external datasets when those markers are sensitive to platform or cohort composition. In contrast, a network-supported signature has greater resilience because its biological meaning is distributed across several coordinated genes (8, 9, 12, 13, 17). As shown in Figure 2, the discovery-layer heatmap provided visual support that the 11-gene signature retained internal redundancy and biological coherence, both of which are favorable for external evaluation.

### The main external analysis showed moderate discrimination between OOHCA-SIRS and sepsis

**Figure 3:**
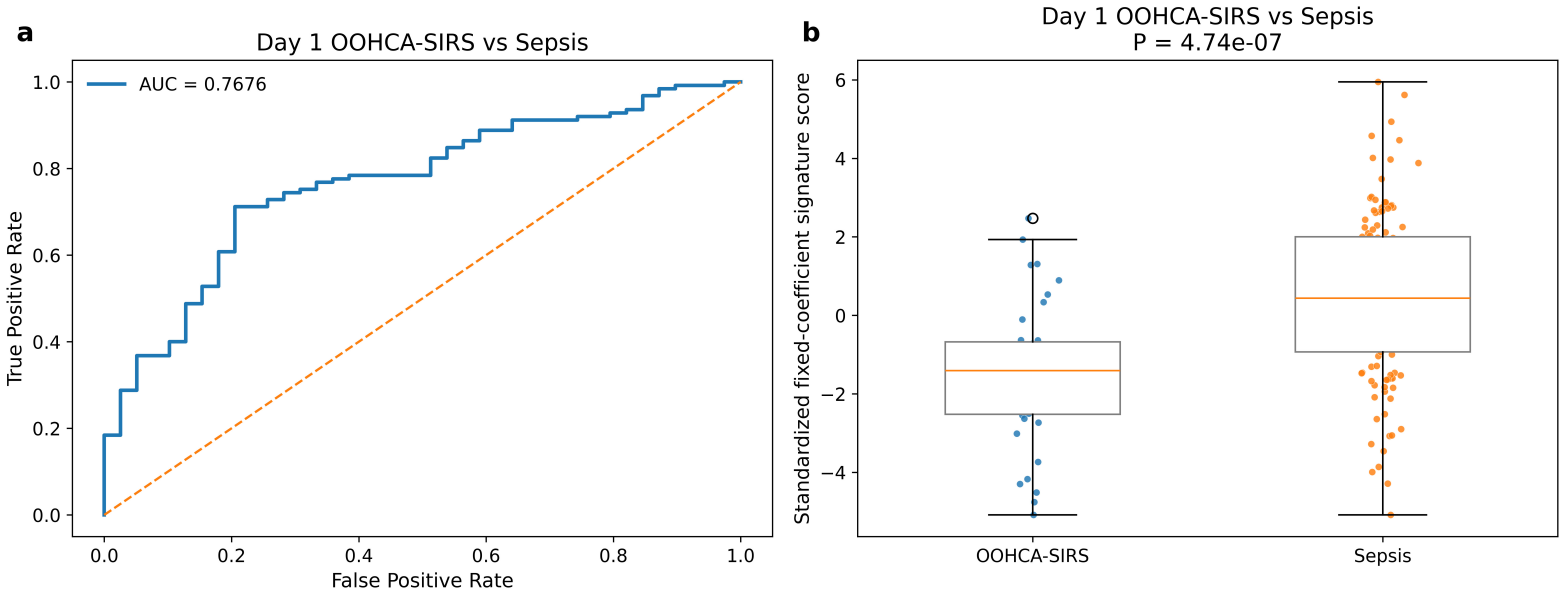
External analysis of the 11-gene signature for Day 1 OOHCA-SIRS vs. sepsis in GSE236713. **(a)** Receiver operating characteristic curve showing the moderate discriminatory performance of the standardized fixed-coefficient 11-gene signature score for Day 1 OOHCA-SIRS vs. sepsis (AUC = 0.7676). **(b)** Distribution of the standardized fixed-coefficient score in the OOHCA-SIRS and sepsis groups shown as boxplots with overlaid individual samples (Mann–Whitney *P* = 4.74 × 10^−7^). OOHCA-SIRS, out-of-hospital cardiac arrest-associated systemic inflammatory response syndrome; AUC, area under the curve.

The main external supportive result was observed in the Day 1 OOHCA-SIRS vs. sepsis comparison in GSE236713. This analysis included 39 OOHCA-SIRS samples and 125 sepsis samples. ROC analysis using the standardized fixed-coefficient score showed moderate discrimination in this setting, with an AUC of 0.7676. Group-wise score distributions differed significantly by the Mann–Whitney *U*-test (*P* = 4.74 × 10^−7^). The corresponding validation statistics, including group counts, signature-gene overlap, AUC, and Mann-Whitney P-value, are summarized in Table 3, and the ROC and score-distribution plots are shown in Figure 3. This result was interpreted as moderate supportive evidence that the score may reflect a sepsis-associated host-response pattern, rather than evidence that it captures a universal host-response state.

**Table 3:** Summary statistics for Day 1 OOHCA-SIRS vs. sepsis validation in GSE236713.

Comparison	n_total	n_sirs	n_sepsis	n_signature_overlap_genes	Auc	Mann–Whitney_*p*
Day1 OOHCA_SIRS vs. Sepsis	164	39	125	11	0.7676	4.74E−07

This table reports the sample size, group counts, number of overlapping signature genes, AUC, and Mann–Whitney *P*-value for the Day 1 OOHCA-SIRS vs. sepsis comparison in GSE236713.

This comparison was selected as the primary validation setting because it tests the signature against a clinically realistic inflammatory control rather than an idealized healthy comparator. OOHCA-SIRS is a stringent comparator because these patients can exhibit substantial systemic stress, inflammation, and organ dysfunction in the absence of infection-driven sepsis biology (17, 21, 22). The observed separation therefore supports the cautious interpretation that the score may reflect a component of infection-associated immune dysregulation rather than a generic critical-illness signal (6, 13, 17, 22). To assess whether the external discrimination was also supported at the gene level, we next visualized the expression structure of the 11-gene panel in the same OOHCA-SIRS vs. sepsis comparison.

### External heatmap analysis supported coordinated multigene directionality in the primary external comparison

**Figure 4:**
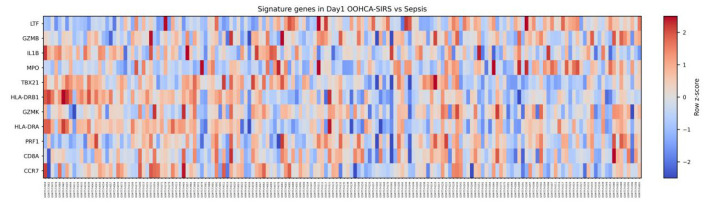
Gene-level heatmap supporting the primary external analysis in GSE236713. Heatmap showing row-scaled expression of the 11-gene signature across Day 1 OOHCA-SIRS and sepsis samples in GSE236713. The coordinated directional pattern across multiple genes indicates that the external analysis signal was supported by multigene structure rather than by a single dominant marker. z-score, within-gene standardized expression value.

An external heatmap from the same OOHCA-SIRS vs. sepsis comparison provided gene-level biological context for the AUC value. Rather than being driven by a single outlying gene, the separation was associated with a coordinated directional shift across the full 11-gene panel. Genes associated with granulocyte-dominant inflammation, including LTF and MPO, were more enriched toward the sepsis side, whereas several lymphoid or antigen presentation–related genes displayed a contrasting distribution across the two groups. This preservation of a broadly compatible directional structure in an independent cohort provided descriptive support that the score retained part of its internal biological architecture beyond the discovery dataset (8, 9, 12). In practical terms, the main external analysis was supported at two descriptive levels: first, by overall discrimination as quantified by ROC analysis; and second, by a gene-level pattern consistent with the underlying biological hypothesis. As shown in Figure 4, the gene-level heatmap supported this multigene structure; together, the score-level discrimination and gene-level pattern provided broader descriptive support than a classifier performance metric alone (13, 17, 22).

### Boundary analysis did not show clear discrimination between abdominal and pulmonary sepsis

**Figure 5:**
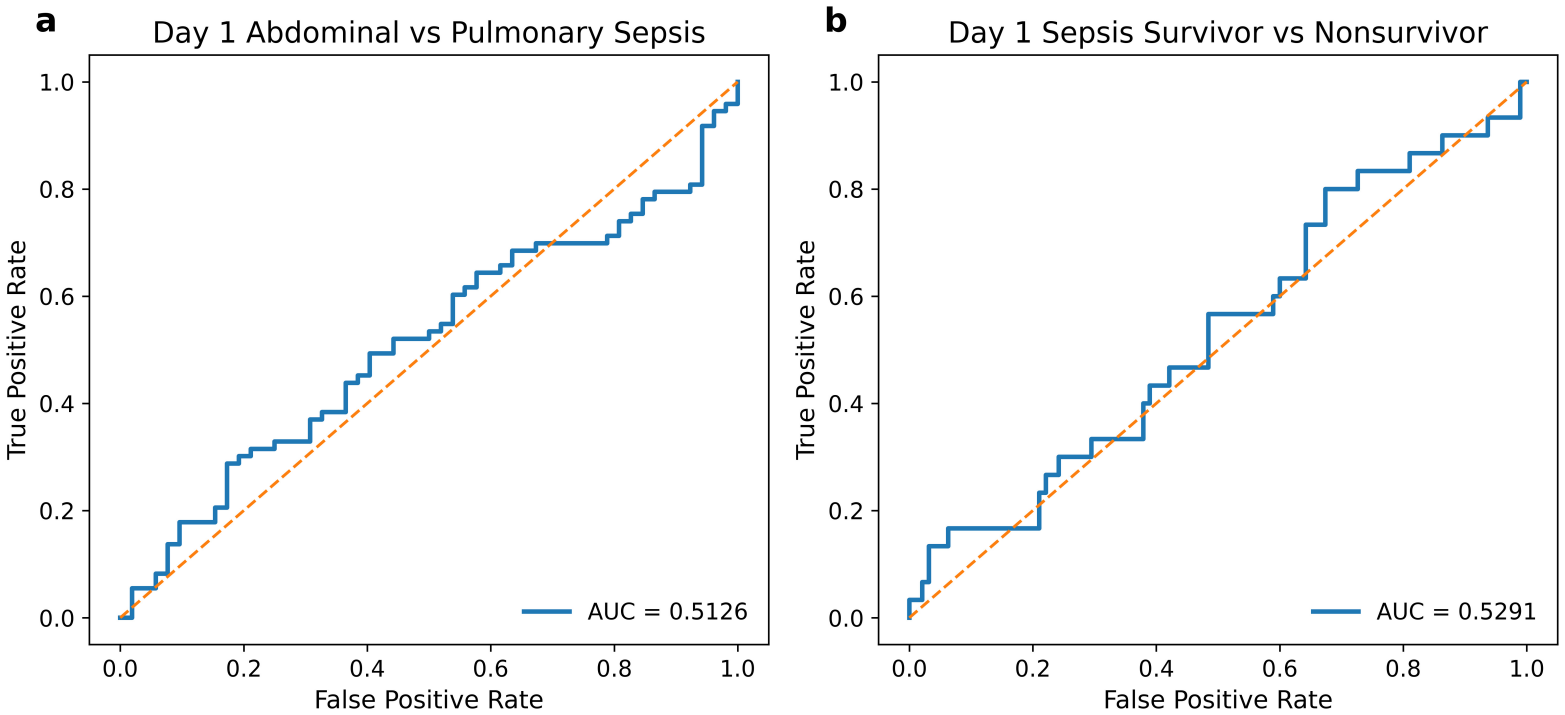
Boundary analyses of the 11-gene signature in GSE236713. **(a)** Receiver operating characteristic analysis of the standardized fixed-coefficient 11-gene signature score for distinguishing Day 1 abdominal sepsis from pulmonary sepsis (AUC = 0.5126). **(b)** Receiver operating characteristic analysis of the standardized fixed-coefficient score for distinguishing Day 1 sepsis survivors from nonsurvivors (AUC = 0.5291). Both analyses showed near-null discrimination under the current Day 1 analysis settings. These findings indicate that the score did not clearly discriminate infection source or survival status in the present dataset, but they should not be interpreted as definitive evidence of source independence or absence of prognostic relevance. AUC, area under the curve.

When the same standardized fixed-coefficient score was applied to Day 1 abdominal vs. pulmonary sepsis in GSE236713, ROC analysis showed near-null discrimination. The AUC was 0.5126, and the groupwise score distributions did not differ significantly according to the Mann–Whitney *U*–test (*P* = 0.8119). The corresponding source-comparison statistics are summarized in Table 4, and the ROC curve for this boundary analysis is shown in Figure 5A. This finding indicates that the score did not show clear source-discriminatory performance under the current Day 1 validation setting. However, this near-null result should not be interpreted as definitive evidence that the signature is universally unrelated to infection source. The observed near-null result may also reflect limitations including subgroup sample size, infection-source annotation granularity, single-time-point measurement, and limited statistical power. Therefore, the result was used to define the current interpretive boundary of the score rather than to make an absolute source-independence claim.

**Table 4:** Summary statistics for Day 1 abdominal vs. pulmonary sepsis validation in GSE236713.

Comparison	*n*_total	n_abdominal	n_pulmonary	n_signature_overlap_genes	Auc	Mann–Whitney_*p*
Day1 abdominal vs. pulmonary sepsis	125	52	73	11	0.5126	0.8119

This table reports the sample size, subgroup counts, number of overlapping signature genes, AUC, and Mann–Whitney *P*-value for the Day 1 abdominal vs. pulmonary sepsis comparison in GSE236713.

### Boundary analysis did not show clear short-term survival discrimination

The Day 1 survival analysis among patients with sepsis in GSE236713 also showed near-null discrimination after standardized fixed-coefficient scoring (AUC = 0.5291; Mann–Whitney *P* = 0.6334). The corresponding survival-comparison statistics are summarized in Table 5, and the ROC curve for this analysis is shown in Figure 5B. This finding indicates that the score did not clearly discriminate survivors from nonsurvivors in this analysis. However, it should not be interpreted as definitive evidence that the signature has no prognostic relevance in other settings. Early mortality in sepsis is influenced by numerous variables, including baseline frailty, pathogen burden, organ-specific injury, hemodynamic instability, comorbidity structure, treatment timing, and endpoint definition. Therefore, the current result should be regarded as a dataset- and endpoint-specific near-null finding rather than as a universal statement about prognostic value.

**Table 5:** Summary statistics for Day 1 survival analysis among patients with sepsis in GSE236713.

Comparison	n_total	n_survived	n_died	n_signature_overlap_genes	auc	Mann–Whitney_*p*
Day1 Sepsis survival	125	95	30	11	0.5291	0.6334

This table reports the sample size, subgroup counts, number of overlapping signature genes, AUC, and Mann–Whitney *P*-value for the Day 1 survivor vs. nonsurvivor comparison in GSE236713.

### Supplementary analysis in GSE54514 remained non-confirmatory

Given the smaller and less balanced sample structure of GSE54514, this cohort was interpreted as supplementary and non-confirmatory. After standardized fixed-coefficient scoring, the Day 1 control vs. sepsis comparison (18 controls and 35 sepsis samples) showed weak directional support but did not reach statistical significance (AUC = 0.6016; Mann–Whitney *P* = 0.2330); it was therefore treated as non-confirmatory. The survivor vs. nonsurvivor comparison included 26 survivors and nine nonsurvivors and did not support a prognostic interpretation (AUC = 0.3761; Mann–Whitney *P* = 0.2821). Therefore, GSE54514 was not used to claim confirmatory external validation, definitive portability, or prognostic performance of the signature.

## Discussion

### A compact signature can preserve the core architecture of sepsis host-response heterogeneity

The present study was motivated by a translational problem that has become increasingly visible in sepsis transcriptomics: the field has generated biologically rich subtype frameworks, but many remain difficult to reduce into portable forms that can be tested or deployed across independent settings (6, 8, 9, 12). Our goal was not to construct a more complex classifier but to derive a compact 11-gene signature directly from an internally coherent subtype structure and then define its meaning through multi-scenario external evaluation. The observation that this score preserved subtype-associated biological organization in the discovery cohort and showed moderate discrimination between OOHCA-SIRS and sepsis in an external cohort suggests that a reduced representation of sepsis-associated host-response biology may retain interpretability and potential relevance. This point is important because translational progress often depends less on maximal dimensionality than on the stability, compactness, and portability of a biologically meaningful signal across analytic environments (13, 14, 17, 22).

### The 11-gene score should be interpreted as a multivariable state index

It is important to note that the 11-gene score should not be interpreted as a simple directional expression scale. The score was derived from a multivariable LASSO-logistic model, and each coefficient represents the mathematical contribution of a gene to the fixed weighted score after joint modeling with the other retained genes. This differs from subtype-level differential expression, which describes the observed mean expression difference for each gene between Subtype_2 and Subtype_1. This distinction is also methodologically important because the coefficients were estimated from the Abdominal_Sepsis vs. GI_Control modeling contrast, whereas the subtype-level expression directions were used to interpret the internal host-response structure.

Within the discovery-layer expression comparison, LTF and MPO showed higher mean expression in Subtype_2 and contributed to the myeloid or granulocyte-associated component of the signature. Several other genes in the panel, including GZMB, PRF1, GZMK, TBX21, CCR7, CD8A, HLA-DRA, and HLA-DRB1, are associated with cytotoxic lymphocyte activity, T cell–related programs, or antigen presentation. However, their coefficient signs should not be equated with their subtype-level expression directions. This distinction is particularly relevant for GZMK and CD8A, both of which had positive coefficients in the fixed score but lower mean expression in Subtype_2 than in Subtype_1. Therefore, the biological interpretation of the signature should be based on the coordinated multigene pattern rather than on assigning a direct functional direction to each coefficient.

This interpretation is consistent with the biological complexity of sepsis, in which heightened innate inflammatory activity may coexist with altered adaptive immune coordination and antigen-presentation programs. The value of the signature therefore lies in its ability to summarize a discovery-derived host-response pattern in compact form, rather than in interpreting each coefficient as an isolated marker of pathway activation or suppression.

### The OOHCA-SIRS comparison represents the main externally supported analysis setting

The main external result in GSE236713 is particularly informative because it clarifies the most plausible real-world context in which the signature may be interpreted. The Day 1 AUC of 0.7676 in the OOHCA-SIRS vs. sepsis comparison provides moderate supportive evidence in a severe inflammatory control setting, rather than in a comparison with healthy controls. Patients with OOHCA-SIRS are critically ill, have systemic inflammation, and frequently have organ dysfunction, but they are not defined by infection-driven sepsis biology. Therefore, a signature that separates these groups may be more likely to reflect infection-associated immune dysregulation than generic critical illness or physiologic stress. This finding supports the more cautious interpretation that the 11-gene panel may reflect a sepsis-associated host-response pattern. From a translational perspective, this is a more meaningful claim than simple control vs. case discrimination because it addresses a boundary that more closely resembles the diagnostic and biological ambiguity encountered in critical care.

### Near-null boundary analyses define the current interpretive limits of the score

The current analyses did not show clear discrimination by infection source or short-term survival under the available Day 1 analysis settings. These near-null findings should be interpreted cautiously because they may be influenced by subgroup size, endpoint heterogeneity, single-time-point measurement, annotation granularity, and limited statistical power. Therefore, the score should not be positioned as a source classifier or early mortality model on the basis of the current evidence. Instead, the present data support its cautious interpretation as an exploratory sepsis-associated host-response score requiring further validation.

### The supplementary cohort exposes the limits of current public data validation

The GSE54514 analyses provided additional context for the main findings but should be interpreted only as supplementary and non-confirmatory. In GSE54514, the control vs. sepsis comparison showed weak directional compatibility, whereas the survivor vs. nonsurvivor comparison did not support a prognostic interpretation. Therefore, this cohort was treated as supplementary and non-confirmatory rather than as decisive external validation.

These findings also highlight a broader challenge in public cohort transcriptomic validation. Differences in sample composition, timepoint distribution, annotation detail, and platform quality can substantially affect the apparent performance of a transcriptomic signature. Therefore, the present results support only a cautious conclusion: the supplementary cohort showed weak directional compatibility in the control vs. sepsis comparison but provided no support for a prognostic interpretation. Further definitive external evaluation will therefore require larger cohorts with harmonized clinical annotation and more consistent sample timing. This is consistent with the trajectory of prior host-response signature studies, which often moved from discovery and public validation to prospective or multicenter validation only after multiple iterative refinements (17, 23–26).

### Limitations and implications for future translational development

Several limitations deserve explicit discussion. First, this study was based on a secondary analysis of public datasets, and heterogeneity in patient enrollment, specimen handling, platform technology, and treatment context could not be eliminated. Second, the primary external analysis provided moderate supportive evidence in the OOHCA-SIRS vs. sepsis comparison, whereas the infection-source and survival analyses were near-null and the GSE54514 analyses were supplementary and non-confirmatory. Therefore, the current evidence should be regarded as exploratory and supportive rather than definitive. Third, the study remained transcriptome-centered and lacked orthogonal confirmation through protein assays, flow cytometry, or prospective clinical sampling.

A further limitation is that GSE154918 could not be incorporated into the formal external analysis framework because a reproducible phenotype–expression linkage could not be established with sufficient stability. This highlights a practical challenge in public omics research: dataset availability does not guarantee suitability for external evaluation. In addition, the score was not further optimized using complex machine-learning pipelines, as the study prioritized biological clarity and external interpretability over maximal performance tuning.

Future work should test the signature prospectively in ICU and emergency populations, evaluate whether it adds value when integrated with protein biomarkers and clinical severity measures, and determine whether it can serve as a practical surrogate for more complex immune-state phenotyping, including single-cell–defined myeloid and lymphoid programs (22, 27–33).

## Conclusion

The present study identified an 11-gene blood transcriptomic score that may reflect a sepsis-associated host-response pattern. The score aligned with the internal discovery-layer host-response structure and showed moderate discrimination between Day 1 sepsis and OOHCA-SIRS in GSE236713. In contrast, it did not show clear discrimination by infection source or short-term survival under the current Day 1 analysis settings. These near-null findings should be interpreted cautiously and should not be construed as definitive evidence of source independence or absence of prognostic relevance. Taken together, the 11-gene score offers a compact and biologically interpretable framework for exploratory sepsis stratification, although its clinical and translational utility warrants further evaluation in larger, prospectively annotated cohorts.

## Data Availability

The original contributions presented in the study are included in the article/Supplementary material, further inquiries can be directed to the corresponding author.
