# Supplementary material for: An 11-gene blood transcriptomic signature reflects a sepsis-associated host-response pattern across public cohorts
Source: Front Med (Lausanne). 2026 Jun 18;13:1844619. doi: 10.3389/fmed.2026.1844619 (PMC13323005; doi:10.3389/fmed.2026.1844619)
Supplement: Supplementary file 1 [file Table_1.docx]

**Supplementary Appendix**

This supplementary appendix contains the 13-feature subtype-discovery evidence, k-selection metrics, stepwise 11-gene signature construction workflow, software environment information, and sensitivity comparison between raw-expression weighted scoring and standardized fixed-coefficient scoring.

## Supplementary Table 1. Definition of the 13 host-response features used for subtype discovery in GSE65682.

| Feature ID | Feature name | Biological axis | Score construction | Module or gene-set source | Role in subtype discovery |
| --- | --- | --- | --- | --- | --- |
| F01 | signature_score | Internal abdominal-sepsis versus GI-control 11-gene signature | Predicted probability from a StandardScaler plus L1 LogisticRegressionCV model trained with Abdominal_Sepsis=1 and GI_Control=0. | Top-20 hub genes filtered by LASSO-logistic selection; final selected genes and coefficients are summarized in the main manuscript, and the stepwise construction workflow is summarized in Supplementary Table 3. | Used as one of 13 z-score scaled features for K-means subtype discovery |
| F02 | GSVA_Neutrophil_Activation | Neutrophil Activation | Mean expression of valid pathway genes after gene-wise z-score standardization across samples; described in script as simplified GSVA/pathway score. | MMP8, LCN2, LTF, BPI, CEACAM8, DEFA4, FCGR3B, ELANE, MPO, CAMP | Used as one of 13 z-score scaled features for K-means subtype discovery |
| F03 | GSVA_Interferon_Response | Interferon Response | Mean expression of valid pathway genes after gene-wise z-score standardization across samples; described in script as simplified GSVA/pathway score. | IFIT1, IFIT2, IFIT3, ISG15, MX1, OAS1, OAS2, RSAD2, IFI44, IFI44L | Used as one of 13 z-score scaled features for K-means subtype discovery |
| F04 | GSVA_T_Cell_Function | T Cell Function | Mean expression of valid pathway genes after gene-wise z-score standardization across samples; described in script as simplified GSVA/pathway score. | IL7R, LTB, MALAT1, MKI67, TRBC1, TRBC2, CD3D, CD3E, PRF1, GZMB | Used as one of 13 z-score scaled features for K-means subtype discovery |
| F05 | GSVA_Antigen_Presentation | Antigen Presentation | Mean expression of valid pathway genes after gene-wise z-score standardization across samples; described in script as simplified GSVA/pathway score. | HLA-DRA, HLA-DRB1, HLA-DPA1, HLA-DPB1, CD74, HLA-DMA, HLA-DMB, CIITA | Used as one of 13 z-score scaled features for K-means subtype discovery |
| F06 | GSVA_Platelet_Coagulation | Platelet Coagulation | Mean expression of valid pathway genes after gene-wise z-score standardization across samples; described in script as simplified GSVA/pathway score. | PF4, PPBP, CLU, F13A1, THBS1, ITGA2B, GP9, TREML1, SPARC, VCL | Used as one of 13 z-score scaled features for K-means subtype discovery |
| F07 | GSVA_Mucosal_Barrier_Defense | Mucosal Barrier Defense | Mean expression of valid pathway genes after gene-wise z-score standardization across samples; described in script as simplified GSVA/pathway score. | OLFM4, LCN2, DEFA4, LTF, BPI, MUC1, MUC20, REG1A, REG3A, DUOX2 | Used as one of 13 z-score scaled features for K-means subtype discovery |
| F08 | IMMUNE_Antigen_Presentation | Antigen Presentation | Mean expression of valid marker genes after gene-wise z-score standardization across samples. | HLA-DRA, HLA-DRB1, HLA-DPA1, HLA-DPB1, CD74, HLA-B, B2M, TAP1 | Used as one of 13 z-score scaled features for K-means subtype discovery |
| F09 | IMMUNE_CD8_T_cells | CD8 T cells | Mean expression of valid marker genes after gene-wise z-score standardization across samples. | CD8A, CD8B, NKG7, CCL5, GZMK, PRF1, CTSW, TRAC | Used as one of 13 z-score scaled features for K-means subtype discovery |
| F10 | IMMUNE_Dendritic_cells | Dendritic cells | Mean expression of valid marker genes after gene-wise z-score standardization across samples. | FCER1A, CST3, HLA-DRA, HLA-DPA1, CLEC10A, CD1C, IRF8, BATF3 | Used as one of 13 z-score scaled features for K-means subtype discovery |
| F11 | IMMUNE_Neutrophils | Neutrophils | Mean expression of valid marker genes after gene-wise z-score standardization across samples. | FCGR3B, CEACAM8, S100A8, S100A9, MMP8, ELANE, LCN2, CAMP | Used as one of 13 z-score scaled features for K-means subtype discovery |
| F12 | IMMUNE_Platelets | Platelets | Mean expression of valid marker genes after gene-wise z-score standardization across samples. | PPBP, PF4, NRGN, TUBB1, GP9, ITGA2B, CLU, SPARC | Used as one of 13 z-score scaled features for K-means subtype discovery |
| F13 | IMMUNE_T_cells | T cells | Mean expression of valid marker genes after gene-wise z-score standardization across samples. | CD3D, CD3E, CD3G, TRBC1, TRBC2, LTB, IL7R, MALAT1 | Used as one of 13 z-score scaled features for K-means subtype discovery |

Note. The signature_score feature represents an internal discovery-layer score dimension included in the 13-feature matrix for subtype discovery. Therefore, discovery-layer subtype alignment was interpreted as internal biological consistency rather than independent validation of the final 11-gene score. External evaluation was performed separately by applying the fixed 11-gene coefficients to independent cohorts without subtype reclustering, feature reselection, model refitting, or coefficient re-estimation.

## Supplementary Table 2. K-selection metrics for the GSE65682 discovery-layer subtype analysis.

| k | silhouette score | inertia | Calinski–Harabasz index | Davies–Bouldin index | cluster size summary | minimum cluster proportion | interpretation |
| --- | --- | --- | --- | --- | --- | --- | --- |
| 2 | 0.3678024789612977 | 675.5723198792398 | 71.85303107099794 | 1.056966512573954 | cluster_0=50;cluster_1=43 | 0.4623655913978494 | Highest silhouette among k=2..6; balanced two-cluster solution. |
| 3 | 0.2791897797501037 | 573.4329061947928 | 49.875964410592864 | 1.3308181827676984 | cluster_0=47;cluster_1=16;cluster_2=30 | 0.1720430107526881 | Alternative k evaluated for sensitivity; not selected by maximum silhouette. |
| 4 | 0.1962181620841991 | 504.3636783219191 | 41.446701606270786 | 1.5987865293315242 | cluster_0=29;cluster_1=16;cluster_2=21;cluster_3=27 | 0.1720430107526881 | Alternative k evaluated for sensitivity; not selected by maximum silhouette. |
| 5 | 0.2003893465507868 | 451.1493393930869 | 36.95608765776144 | 1.4840638351024145 | cluster_0=16;cluster_1=18;cluster_2=25;cluster_3=19;cluster_4=15 | 0.1612903225806451 | Alternative k evaluated for sensitivity; not selected by maximum silhouette. |
| 6 | 0.2080271257971661 | 406.834436054993 | 34.30801224200307 | 1.364712035582862 | cluster_0=15;cluster_1=18;cluster_2=3;cluster_3=14;cluster_4=19;cluster_5=24 | 0.032258064516129 | Alternative k evaluated for sensitivity; not selected by maximum silhouette. |

## Supplementary Table 3. Stepwise construction process of the 11-gene signature.

| Step | Analytical component | Input | Selection or ranking rule | Output |
| --- | --- | --- | --- | --- |
| 1 | Discovery subtype definition | GSE65682 discovery-layer samples | A fixed 13-feature host-response matrix from 93 GSE65682 discovery-layer samples was scaled using StandardScaler and clustered using K-means. Candidate k values from 2 to 6 were evaluated using silhouette score, inertia, Calinski–Harabasz index, Davies–Bouldin index, and cluster balance. k = 2 yielded a silhouette score of 0.3678 and inertia of 675.5723, with cluster sizes of 50 and 43. After label harmonization, the assignments defined Subtype_1 n = 43 and Subtype_2 n = 50. | Subtype_1 n = 43; Subtype_2 n = 50 |
| 2 | Disease-comparison differential-expression evidence | GSE65682 Abdominal_Sepsis and GI_Control samples | Abdominal_Sepsis versus GI_Control DEG analysis; Benjamini–Hochberg adjusted P value < 0.05 and absolute log2 fold change ≥ 1.0 | 304 significant DEG-supported candidate genes |
| 3 | STRING-PPI hub prioritization | 304 significant DEG-supported candidate genes | STRING network API; Homo sapiens species 9606; required_score = 400; genes ranked by node degree, with weighted degree retained as a secondary network quantity; top 20 degree-ranked hub genes selected | Top 20 STRING-PPI hub candidates |
| 4 | Penalized modeling | Top 20 STRING-PPI hub candidates | Abdominal_Sepsis = 1 and GI_Control = 0; StandardScaler + LogisticRegressionCV; L1 penalty; saga solver; 5-fold StratifiedKFold; roc_auc scoring; random_state = 42; max_iter = 5000 | LASSO coefficient estimates |
| 5 | Final gene retention | LASSO-logistic model output | Nonzero coefficient rule | 11 retained genes |
| 6 | Score construction | 11 retained genes and fixed coefficients | Weighted sum of cohort-specific standardized gene expression values and fixed discovery-derived coefficients | Standardized fixed-coefficient 11-gene signature score |
| 7 | External evaluation | GSE236713 and GSE54514 | Cohort-specific gene-level reconstruction followed by cohort-specific StandardScaler transformation of the 11 retained genes; no reclustering; no feature reselection; no model refitting; no coefficient re-estimation | Standardized fixed-score external analysis |

## Supplementary Table 4. Software environment and package versions.

| Software/package | Version |
| --- | --- |
| Python | 3.10.11 |
| pandas | 2.3.3 |
| numpy | 1.26.4 |
| scipy | 1.15.3 |
| scikit-learn | 1.7.2 |
| matplotlib | 3.10.8 |

Supplementary Table 5. Sensitivity comparison between raw-expression weighted scoring and standardized fixed-coefficient scoring.

| cohort | comparison | raw_auc | raw_mannwhitney_p | standardized_auc | standardized_mannwhitney_p | direction_changed | conclusion_changed | interpretation |
| --- | --- | --- | --- | --- | --- | --- | --- | --- |
| GSE236713 | Day1 OOHCA-SIRS vs sepsis | 0.7618 | 8.29E-07 | 0.7676 | 4.74E-07 | False | False | Main conclusion unchanged; standardized scoring retained moderate discrimination. |
| GSE236713 | Day1 abdominal vs pulmonary sepsis | 0.5087 | 0.8707 | 0.5126 | 0.8119 | False | False | Main conclusion unchanged; standardized scoring remained near null. |
| GSE236713 | Day1 sepsis survivor vs nonsurvivor | 0.5267 | 0.6625 | 0.5291 | 0.6334 | False | False | Main conclusion unchanged; standardized scoring remained near null. |
| GSE54514 | Day1 control vs sepsis | 0.5984 | 0.2481 | 0.6016 | 0.2330 | False | False | Weak directional support in the control versus sepsis comparison; non-confirmatory. |
| GSE54514 | Day1 survivor vs nonsurvivor | 0.4274 | 0.5334 | 0.3761 | 0.2821 | False | False | Non-confirmatory; no prognostic claim supported. |

Note. Raw-expression weighted scores are shown only as a sensitivity comparator from the prior audit and are not retained as the main external-analysis result. The standardized fixed-coefficient score is the revised main scoring convention.
